# A randomized controlled simulation trial comparing video-assisted with telephone-assisted and unassisted cardiopulmonary resuscitation performed by non-healthcare university students

**DOI:** 10.1038/s41598-023-42131-z

**Published:** 2023-09-11

**Authors:** Vivien Szöllősi, Balázs Horváth, Dániel Németh, Henrietta Bánfai-Csonka, József Betlehem, Bálint Bánfai

**Affiliations:** 1 Institute of Emergency Care, Pedagogy of Health and Nursing Sciences, University of Pécs Faculty of Health Sciences, Vörösmarty Street 4, 7621 Pécs, Hungary; 2 National Ambulance Service, Szombathely, Hungary; 3 Doctoral School of Health Sciences, University of Pécs Faculty of Health Sciences, Vörösmarty Street 4, 7621 Pécs, Hungary; 4 Department of Emergency Medicine, Clinical Centre, University of Pécs, Ifjúság Street 13., 7624 Pécs, Hungary

**Keywords:** Cardiology, Health care, Medical research

## Abstract

Our randomized controlled simulation study aimed to compare the CPR quality, time-related factors, attitude and self-assessment of non-healthcare university students (aged 18–25) compared video-assisted (V-CPR, n = 50) with telephone-assisted (T-CPR, n = 49) and unassisted (U-CPR, n = 48) CPR in a simulation setting. Regarding to chest compression depth, no difference was found between the three groups (p = 0.065): 41.8 mm, SD = 9.9 in the V-CPR; 35.9 mm, SD = 11.6 in the T-CPR; and 39.4 mm, SD = 15.6 in the U-CPR group. The mean chest compression rate was the best in the V-CPR group (100.9 min^−1^, SD = 17.1) which was superior to the T-CPR (82.4 min^−1^, SD = 35.4; p = 0.005), and the U-CPR (84.2 min^−1^, SD = 30.6; p = 0.013) groups. The overall proportion of correct hand position was the highest in the V-CPR group (48, 96%), compared to the T-CPR (28, 57.1%; p = 0.001), and the U-CPR (34, 70.8%; p = 0.001) groups. V-CPR led to a delay in the time to the first chest compression compared with the U-CPR group (77.5 s, SD = 19.2 vs. 31.3 s, SD = 13.3, p < 0.001). Although V-CPR technology holds the potential to improve overall CPR quality, the importance of appropriate chest compression depth should be emphasized in training for laypeople and dispatchers, as well. Our study was registered at ClinicalTrials.gov (NCT05639868, 06/12/2022).

## Introduction

Sudden cardiac arrest is a major public health problem worldwide and it is one of the leading causes of death in industrialized countries^1^. Survival rates after out-of-hospital cardiac arrest (OHCA) are very low^2^. Early recognition and activation of the Emergency Medical Services (EMS) are essential to improve outcomes^3^. When cardiopulmonary resuscitation (CPR) is provided rapidly, survival rates are higher and neurological outcomes are better^4^. There is a quite large variation in bystander CPR rates between countries (13–82%)^5^.

In the majority of the cases, the bystanders who call the ambulance are laypeople, for whom the identification of OHCA is difficult without experience. Therefore, EMS dispatchers play an important role to recognize cardiac arrest and give help to the lay first responder via telephone CPR (T-CPR) which improves survival rates^6^. However, the audio-only method has several limitations, such as the dispatcher can not see the victim and the bystander, and can give instructions only without visualization of the situation.

The current technology allows the live video connection between the scene and the dispatcher which provides the opportunity for video-assisted CPR (V-CPR). In some prior studies, V-CPR showed higher efficacy in some aspects of chest compressions than T-CPR^7,8^. These V-CPR systems were associated with favorable outcomes in adult patients with OHCA and showed improvement in chest compression parameters^9–11^. However, despite improvement in overall CPR performance, participants could not reach guidelines standards related to chest compression depth^7,8^. In addition, creating video connection can take more time than an audio-only connection, and technical aspects are also important (e.g. camera position, video quality, environmental factors, quality of the signal, etc.)^11–14^. Therefore, despite promising results, there are further gaps in the research evidence related to V-CPR so prior studies suggested further investigations. The effectiveness of CPR is essential, but time-related factors and the attitude of the lay responders are also important.

The aim of this study was to compare the CPR quality (chest compression depth and rate, hand position), the CPR-related time intervals (time of check the breathing, time to call the ambulance, time to the first chest compression), and the attitude and self-assessment of lay responders comparing unassisted CPR (U-CPR), T-CPR and V-CPR. We hypothesized that the quality and effectiveness of the V-CPR method are superior to the quality of U-CPR and T-CPR in a simulation setting.

## Methods

### Study design

A randomized controlled, superiority, simulation trial was performed. The Consolidated Standards of Reporting Trials (CONSORT) reporting guideline was followed^15^. The research complies with all relevant ethical regulations. The study was conducted according to the principles of the Declaration of Helsinki (the most recent version established at the 64th WMA General Assembly, Fortaleza, Brazil, October 2013) and in accordance with the Medical Research Involving Human Subjects Act (WMO). The study protocol was approved by the Institutional Ethics Committee of the University of Pécs (approval number: PTE/87175-1/2022). All participants received detailed information about the research and signed the declaration of informed consent if they agreed to the research conditions and were willing to participate. Participants were informed of their right to quit at any point during the study with no personal consequences.

Our study was registered at ClinicalTrials.gov (registration number: NCT05639868; date of first registration: 06/12/2022).

### Participants

University students from non-healthcare areas were recruited in our study from the Eötvös Lóránd University, Savaria University Centre, Szombathely, Hungary. The study was conducted between December 2022 and January 2023. Eligible for inclusion were university students. A written form was sent via email and students were asked to contact the trial manager if they were willing to participate. Participants who were not capable to perform CPR (e.g. physical or mental impairment) were excluded from the study. Participants did not receive any compensation for their participation.

### Randomization and blinding

Participants were randomly assigned into three different groups before the assessment: U-CPR; T-CPR; and V-CPR. During randomization, the permuted block technique was used with a 1:1:1 ratio. After randomization, study participants were informed about their allocated group, but were blinded to the purpose of the study. The dispatchers, assessors and the operator were informed about their tasks in the different groups but were blinded to the study design and the outcomes.

### Intervention

Participants were accompanied into a room by a study operator who was equipped with a smartphone (iPhone 2013, Apple Inc., USA). Based on a prior study, video quality had no significant impact on the evaluation of CPR performance^14^. Therefore, we thought using this type of smartphone has no impact on the reliability of our study. A CPR manikin connected with the AMBU CPR software (AMBU A/S, Baltorpbakken 13, DK 2750 Ballerup, Denmark) was pre-installed in the room which represented the victim. In every situation, the study participants had the following information before entering the room: „This is a simulated emergency situation. You can hear some noise from this room. You are entering the room and you can find a person who is lying on the floor. Perform appropriate first aid based on your best knowledge and skills. You will be not alone, a study operator equipped with a smartphone will be also inside. Contact the study operator if you think calling the ambulance is necessary. The operator is able to call the ambulance correctly, however, she will not communicate or help you in any further way.” Further initial instructions were given related to the different groups: V-CPR group participants were informed that the dispatcher will be able to see the environment and the performance during first aid via the video call so they should follow the instructions; T-CPR group members were informed that the dispatcher will give audio instructions related to the first aid process; U-CPR group participants were informed that if they would like to call the ambulance they should ask the operator to do that so their only task will be performing first aid.

During the scenario, the participants should perform the initial examinations (check the safety of the environment, check the response and the breathing of the victim), give instructions to the study operator to call the ambulance and start chest compressions. Related to the duration of the intervention, the clock started when the participant entered the room. After that, every participant should perform chest compression-only CPR for 2 min (because based on the current guidelines changing the rescuer during CPR is recommended every 2 min)^16^. After 2 min of chest compressions, the study operator stopped the scenario. Based on the above-mentioned information, the scenario started when the participant entered the room and was terminated by the study operator after 2 min of CPR.

Another room was set up for the dispatcher with a smartphone (for audio communication; iPhone SE 2020, Apple Inc., USA) and a tablet (for video communication; iPad Air 8th Gen, Apple Inc., USA). Two dispatchers were involved in the study who were familiar and experienced with the Hungarian emergency dispatch system and were informed about the study methods. In the U-CPR group, the dispatcher gave the information to the caller that they should perform chest compressions until the ambulance arrives. After that, the call ended and no further instructions were received from the dispatcher. In the T-CPR and V-CPR groups, the dispatcher gave instructions related to high-quality chest compressions based on the current ERC guidelines^16^. In the T-CPR group, the Hungarian standardized T-CPR protocol was used^17^, however, it was shortened and optimized for our study (e.g. no instructions about breathing patient, or no ask for automated external defibrillator (AED) because these were not in the focus of our study) (Supplementary Material, Supplementary Fig. S1). Since there is no standardized V-CPR protocol in Hungary, the standardized T-CPR protocol was used in the V-CPR group but was expanded with the live video picture better reflecting on the live-video possibilities (e.g. the opportunity to give relevant feedback on the hand position or other chest compression related factors). In addition, the standardized T-CPR protocol was modified/optimized for the V-CPR technology at some points (e.g. the „Is the patient laying on his/her back?” question was not necessary in the V-CPR group because the dispatcher could see the patient’s position without asking the lay responder) (Supplementary Material, Supplementary Fig. S2). In the T-CPR and V-CPR groups, counting in every 30 s (for 10 s) was given by the dispatcher. In addition, further encouragement (e.g. “faster”, “harder”, etc.) was received in the V-CPR group after counting to motivate participants. In the V-CPR group, common mistakes (e.g. incorrect method to check breathing, incorrect hand position, incorrect chest compression depth or rate, etc.) were fixed by the dispatcher immediately as there were identified. This latter opportunity was not available in the U-CPR group and the T-CPR group due to the lack of live video picture. During the video call, the dispatcher gave instructions to the operator related to the camera position. The opposite side of the lay responder has chosen which was the most appropriate based on a prior study^13^. Apart from that, there was no communication between the operator and either the dispatcher or the study participants.

During the scenario, the smartphone and the tablet were linked to the internet via secure WiFi (bandwidth 1000 mbits^−1^). The video calls were established using FaceTime application (Apple Inc., USA). For testing feasibility and reliability, test calls were established before the scenarios by the study operator and the dispatchers.

### Data collection

The quality of chest compressions (depth and rate) were measured by the AMBU CPR software and were analyzed retrospectively based on the recorded reports. For calculating proportions of correct chest compression parameters, software recorded data were dichotomized to „correct” (within the intervals described in the guidelines) and „incorrect” (outside the intervals described in the guidelines) categories. Scenarios were also video recorded and analyzed by assessors who were emergency professionals (two paramedics) familiar with Basic Life Support (BLS) education who are familiar with CPR quality assessment. For recording, a camera (Nikon D750, Nikon Corporation, Tokyo, Japan) with a resolution of 1920 × 1080 pictures was pre-installed on a tripod in the room. The camera picture covered the room entrance, the manikin and the activity of the participants. At this latter point, correct hand position during CPR, time of check the breathing, time to call the ambulance and time to the first chest compression were measured. For time-related data, the clock was started when the participants entered the room and stopped after 2 min of chest compressions.

Sociodemographic data (age, body weight and height, prior first aid training, prior real-life CPR experience) were collected by a study assistant immediately after the scenario. Collected data were anonymized. In addition, self-assessment in all groups, and experiences and attitudes related to dispatcher-assisted CPR in T-CPR and V-CPR groups were also measured by filling out an online survey after the scenario (5 items for U-CPR; 6 items for T-CPR and V-CPR). For filling out the survey, a tablet (iPad Air 8th Gen, Apple Inc., USA) was used with the assistance of a study operator. A 4-point Likert-scale was used in the following questions: (1) “How did you feel during the scenario?”; (2) “How calm were you during the scenario?”; (3) “Did you perform CPR in the right way?”; (4) “Were the dispatcher’s instructions useful for you during the scenario?”; (5) “Would it be useful to get instructions from the dispatcher during the scenario?”; (6) “How motivating was it to communicate with the dispatcher during the scenario?”. A “Yes/No” answer could be given to the following question: “Would you use the V-CPR method in a real cardiac arrest situation?”.

### Outcomes

The primary outcome of the study was the quality of chest compressions, measured by the mean depth. The evaluation of data was based on the current ERC guidelines (depth of 50–60 mm)^16^.

The secondary outcome of the study was chest compression quality expressed by rate (min^−1^), and correct hand position. Evaluation of data was based on the current ERC guidelines (rate of 100–120 min^−1^; hand on the lower half of the sternum)^16^. A further secondary outcome was the evaluation of time-related factors such as the time of check the breathing, time from the start of the scenario to calling the ambulance and time from the start of the scenario to the first chest compressions. Time factors were measured and assessed based on video records.

Furthermore, the participant-centered secondary outcome was the evaluation of self-assessment and attitude toward dispatcher-assisted CPR. Data collection and analysis were based on the online survey filled out by the participants immediately after the scenario.

### Sample size calculation

Sample size was calculated to detect a 5 mm difference in chest compression depth between the groups based on prior studies^18,19^. With a power of 80%, an α of 5% and a β of 0.2, we calculated 36 participants per group. To deal with the possible drop-out rate, 50 participants per group (a total of 150) were recruited for the study.

### Statistics

To describe the sample, descriptive statistics were used. Study parameters were assessed for normal distribution and reported as numbers (percentages) and means (SDs). Study parameters were assessed for normality by using Shapiro–Wilk test which indicated normal distribution. Continuous variables were compared using ANOVA and Bonferroni post hoc test as appropriate. Categorical variables were compared using Chi-square test or Fisher’s-exact test as appropriate. A two-tailed p-value of < 0.05 was considered to be statistically significant. Statistical analysis was conducted using SPSS 24.0 (Statistics Package for Social Sciences, Chicago, IL, USA).

## Results

### Participants flow, recruitment, and baseline characteristics

**Figure 1:**
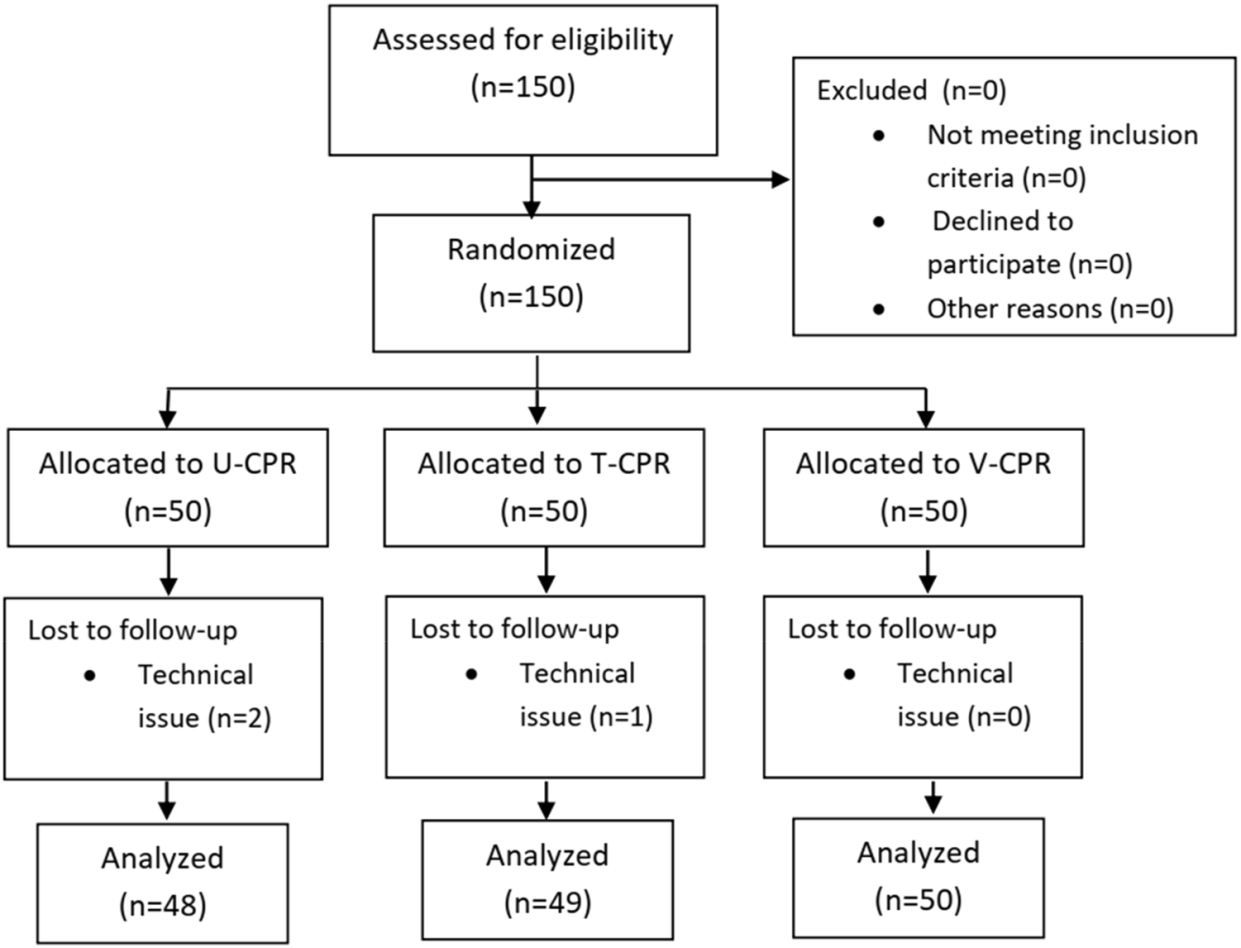
CONSORT diagram. U-CPR, unassisted cardiopulmonary resuscitation; T-CPR, telephone-assisted cardiopulmonary resuscitation; V-CPR, video-assisted cardiopulmonary resuscitation.

In total, 207 emails were sent to the university students (this was the total number of the students on the campus). One hundred and fifty of them responded that they would like to participate our study, so 150 participants were assessed for eligibility. None of the participants were excluded before the randomization. Therefore, 150 participants were randomized: 50 to the V-CPR group, 50 to the T-CPR group, and 50 to the U-CPR group. Three of the participants were excluded (two from the U-CPR group and one from the T-CPR group) because of a technical issue during data collection [the CPR software did not record data]). After exclusions, the total number of participants was 147, respectively. The study flowchart is visible in Fig. 1.

Most of the participants were female (103, 70.1%). The mean age was 20.3 years (SD = 1.4), and the mean BMI was 23.1 kg/m^2^ (SD = 4.3). Of all participants, 130 participated in prior resuscitation training, but at the same time, none of the participants had real-life CPR experience before. There were no significant differences between the baseline characteristics of the participants between the three groups. The main characteristics of the participants are shown in Table 1.

**Table 1:** Basic characteristics of the participants.

Characteristics	In total	U-CPR	T-CPR	V-CPR
Participants (%)	147 (100)	48 (100)	49 (100)	50 (100)
Women (%)	103 (70.1)	33 (68.8)	35 (71.4)	35 (70)
Mean age (SD), years	20.3 (1.4)	20.4 (1.1)	20.6 (1.4)	20.1 (1.5)
Mean body weight (SD), kg	68.4 (15.7)	67.7 (17.6)	68.2 (13.7)	69.2 (15.9)
Mean body height (SD), cm	170.9 (9.2)	169.8 (9.7)	171.6 (8.9)	171.3 (9.1)
Mean BMI, kg/m^2^	23.1 (4.3)	23.1 (4.5)	22.6 (3.2)	23.3 (4.9)
Prior first aid training (%)	130 (88.4)	41 (85.4)	46 (93.9)	43 (86)
Real-life CPR experience (%)	0 (0)	0 (0)	0 (0)	0 (0)

U-CPR, unassisted cardiopulmonary resuscitation; T-CPR, telephone-assisted cardiopulmonary resuscitation; V-CPR, video-assisted cardiopulmonary resuscitation; SD, standard deviation; BMI, body mass index.

### Primary outcomes

In the primary outcome, the mean chest compression depth was 41.8 mm (SD = 9.9) in the V-CPR group, 35.9 mm (SD = 11.6) in the T-CPR group, and 39.4 mm (SD = 15.6) in the U-CPR group. There was no significant difference between the three groups (p = 0.065). Although there were some participants in each group who reached the appropriate chest compression depth but based on the overall data, the mean chest compression depth did not comply with the current ERC guidelines (depth of 50–60 mm). Results are available in Table 2.

**Table 2:** Chest compression performance based on the depth, rate, and correct hand position.

	Group			p-value			
	V-CPR (n = 50)	T-CPR (n = 49)	U-CPR (n = 48)	Overall	V-CPR vs. T-CPR^a^	V-CPR vs. U-CPR^a^	T-CPR vs. U-CPR^a^
Mean chest compression depth (SD), mm	41.8 (9.9)	35.9 (11.6)	39.4 (15.6)	0.065	–	–	–
Proportion of correct chest compression depth (%)	8 (16)	4 (8.2)	6 (12.5)	0.492	–	–	–
Mean chest compression rate (SD), min^−1^	100.9 (17.1)	82.4 (35.4)	84.2 (30.6)	**0.002**	**0.005**	**0.013**	1.000
Proportion of correct chest compression rate (%)	26 (52)	14 (28.6)	6 (12.5)	** < 0.001**	–	–	–
Proportion of correct hand position at the start (%)	26 (52.0)	28 (57.1)	34 (70.8)	0.146	–	–	–
Proportion of correct hand position at the end (%)	48 (96.0)	28 (57.1)	34 (70.8)	**0.028**	–	–	–
Proportion of effective chest compression in all aspects together (%)	5 (10)	2 (4.1)	1 (2.1)	0.282	–	–	–

U-CPR, unassisted cardiopulmonary resuscitation; T-CPR, telephone-assisted cardiopulmonary resuscitation; V-CPR, video-assisted cardiopulmonary resuscitation; SD, standard deviation.

Significant values are in bold.

^a^Post hoc analysis (Bonferroni).

### Secondary outcomes

Related to further chest compression quality parameters, the mean chest compression rate was the best in the V-CPR group (100.9 min^−1^, SD = 17.1) which was superior to the T-CPR (82.4 min^−1^, SD = 35.4; p = 0.005), and the U-CPR (84.2 min^−1^, SD = 30.6; p = 0.013) groups (Table 2). Although there were participants in each group who conformed to the ERC guidelines related to the chest compression rate of 100–120 min^−1^, the highest rate of correct performance was in the V-CPR group. The overall proportion of correct hand position was the highest in the V-CPR group (48, 96%), compared to the T-CPR (28, 57.1%; p = 0.001), and the U-CPR (34, 70.8%; p = 0.001) groups. Only a minority of the participants were able to perform high-quality chest compressions assessing all aspects together. Additional results related to CPR performance are shown in Table 2.

As further secondary outcomes, time-related factors are shown in Table 3. We found a significant difference between the three groups related to the mean time of check the breathing (p = 0.002): 11.7 s (SD = 4.7) in the V-CPR group, 9.8 s (SD = 3.4) in the T-CPR group, and 8.1 s (SD = 6.5) in the U-CPR group. The mean time to call the ambulance was 10.7 s (SD = 13.8) in the V-CPR group, 17.6 s (SD = 13.8) in the T-CPR group, and 21.3 s (SD = 10.3) in the U-CPR group. The difference between V-CPR and T-CPR (p = 0.025), and V-CPR and U-CPR (< 0.001) was significant. Mean time to the first chest compression was superior in the V-CPR group (77.5 s, SD = 19.2) to the T-CPR group (86.1 s, SD = 17.5; p = 0.039), but was inferior to the U-CPR group (31.3 s, SD = 13.3; p < 0.001).

**Table 3:** Time-related factors during the scenario (time of check the breathing, time to call the ambulnace and time to the first chest compression.

	Group			p-value			
	V-CPR (n = 50)	T-CPR (n = 49)	U-CPR (n = 48)	Overall	V-CPR vs. T-CPR^a^	V-CPR vs. U-CPR^a^	T-CPR vs. U-CPR^a^
Mean time of check the breathing (SD), sec	11.7 (4.7)	9.8 (3.4)	8.1 (6.5)	**0.002**	0.176	**0.001**	0.252
Mean time to call the ambulance (SD), sec	10.7 (13.8)	17.6 (13.8)	21.3 (10.3)	** < 0.001**	**0.025**	** < 0.001**	0.515
Mean time to the first chest compression (SD), sec	77.5 (19.2)	86.1 (17.5)	31.3 (13.3)	** < 0.001**	**0.039**	** < 0.001**	** < 0.001**

U-CPR, unassisted cardiopulmonary resuscitation; T-CPR, telephone-assisted cardiopulmonary resuscitation; V-CPR, video-assisted cardiopulmonary resuscitation; SD, standard deviation.

Significant values are in bold.

^a^Post hoc analysis (Bonferroni).

For participant-centered secondary outcomes, no differences between the attitude and self-assessment were found between the three groups based on the self-reported questionnaire. In every group, more than 90% of the participants would use the V-CPR method in a real-life CPR situation. Results related to the attitude and self-assessment are shown in more detail in Table 4.

**Table 4:** Attitude and self-assessment related to dispatcher-assisted CPR. In every case, a 4-point Likert-scale was used (1 was the most negative answer, 4 was the most positive answer).

Question		Group			p-value
		V-CPR (n = 50)	T-CPR (n = 49)	U-CPR (n = 48)	
How did you feel during the scenario?	Mean (SD)	3.4 (0.7)	3.5 (0.6)	3.3 (0.8)	0.401
How calm were you during the scenario?	Mean (SD)	3.3 (0.6)	3.2 (0.8)	3.0 (0.8)	0.144
Did you perform CPR in the right way?	Mean (SD)	3.1 (0.7)	3.3 (0.7)	3.0 (0.6)	0.265
Were the dispatcher’s instructions useful for you during the scenario?	Mean (SD)	3.8 (0.4)	3.6 (0.7)	–	0.101
Would it be useful to get instructions from the dispatcher during the scenario?	Mean (SD)	–	–	3.3 (0.8)	–
How motivating was it to communicate with the dispatcher during the scenario?	Mean (SD)	3.7 (0.4)	3.7 (0.6)	–	0.412
Would you use the V-CPR method in a real cardiac arrest situation?	Ratio of “Yes” answers (%)	48 (96)	45 (91.8)	46 (95.8	0.588

U-CPR, unassisted cardiopulmonary resuscitation; T-CPR, telephone-assisted cardiopulmonary resuscitation; V-CPR, video-assisted cardiopulmonary resuscitation; SD, standard deviation.

## Discussion

In this randomized controlled simulation study, we measured chest compression effectiveness (rate, depth and hand position) and time-related factors (time of check the breathing, time to call the ambulance, and time to the first chest compression) in a cardiac arrest scenario comparing V-CPR, T-CPR and U-CPR groups. In addition, we collected data about CPR self-assessment and attitudes toward V-CPR technology. Since the effectiveness of V-CPR is affected by several factors, we tried to give a wide picture of the topic (CPR effectiveness, time-related factors, attitude and self-assessment). Our results showed that V-CPR method performed by lay responders could lead to higher-quality chest compressions compared to T-CPR and U-CPR. However, V-CPR instructions were not enough to achieve high-quality chest compression depth related to the ERC guidelines in the majority of the cases^16^. Time-related factors such as time to the first chest compression were longer in V-CPR group than in U-CPR group. No differences were found in attitude and self-assessment comparing the V-CPR group with T-CPR and U-CPR groups. In addition, the overall attitude was high about the feasibility of the V-CPR technology based on the results of the self-reported questionnaire.

Early recognition of OHCA, activation of the EMS, and performing high-quality CPR can improve survival rate which can be supported by dispatcher-assisted CPR^1,16^. Prior studies showed that dispatcher instructions during CPR could improve the quality of lay responder’s performance^7^. Video communication is a daily part of our life and can be involved successfully in emergency care, as well^12^. V-CPR could be able to deal with the limitations of T-CPR (visibility of the environment, the victim and the lay responder), however, an appropriate and stable background in technology (e.g. a capable system that allows the live video-based connection between the dispatcher and the caller, adequate signal, acceptable environmental factors) and human factors (e.g. trained dispatchers, lay responders who are familiar with the usage of V-CPR system) are required^13,14,20^. Since V-CPR technology is not implemented in Hungary yet, we used a modified version of the standardized T-CPR protocol. This fact can limit our results because T-CPR and V-CPR instructions can vary in real life. However, we tried to optimize the protocol for V-CPR as much as possible.

In our study, participants performed chest compression-only CPR because it is the recommended protocol during dispatcher-assisted resuscitation in Hungary^17^, and it is more effective based on a prior study than conventional (compression-ventilation) CPR^21^, as well. Chest compression effectiveness was the highest in the V-CPR group. However, despite real-time feedback based on the live video picture, no difference was found related to the chest compression depth between the V-CPR and other groups which indicates one of the limitations of the V-CPR technology. In addition, the overall mean results did not conform with the ERC guidelines related to the chest compression depth of 50–60 mm (in neither group)^16^. Taking into account the participant’s good physical ability which should have allowed for good performance, it is unclear why these poor results related to chest compression depth were obtained. It can be caused by several factors, e.g. low motivation, limitations in the dispatcher’s instructions, low realism of the scenario, and/or instructions that were not clearly audible to the participants. Furthermore, we did not use any CPR feedback device during the scenario, although it is able to improve the quality of CPR, so it can be another explanation of our results^22^. Chest compression rate and correct hand position were superior in the V-CPR group to the T-CPR and U-CPR groups. Although there were participants in each group who conformed to the ERC guidelines related to the chest compression rate of 100–120 min^−1^^16^, the highest rate of participants performing correctly was in the V-CPR group. It can be explained by the real-time feedback from the dispatcher based on the live video connection (the negative results of the T-CPR group—despite vocal instructions—is unknown). This latter point also explains the highest proportion of correct hand position in the V-CPR group (due to the possibility of correction during the process). Some prior studies had similar results on CPR effectiveness^22–24^. Another study had also similar findings, however, their participants were able to achieve the appropriate compression depth in all groups^18^.

Analyzing data from another perspective, we measured time-related parameters. The time of check the breathing was the longest in the V-CPR group. The reason for this result could be that the dispatcher fixed some problems in many cases and gave real-time feedback based on the video picture (e.g. participants did not tilt the head back during the procedure of check the breathing). Participants in the V-CPR group called the ambulance earlier than in the T-CPR and U-CPR groups. It can be explained with the prior information that they will be able to communicate with the dispatcher via a video call if they need some support. Time to the first chest compression was shorter in the V-CPR group than in the T-CPR group, however, was longer than in the U-CPR group. The difference between V-CPR and T-CPR can be explained with the more clear instructions by the dispatchers: in the V-CPR group, dispatchers could see the situation and were able to give adequate prompts, and it was not necessary to ask the lay rescuer for any feedback which could decrease the delays during the process. In addition, the difference between the V-CPR and U-CPR groups can be explained by the fact, that the process is faster (though not necessarily more efficient) when lay rescuers can do the process without any external instructions. Based on a systematic review, dispatcher-assisted CPR demonstrated inferior outcomes compared to unassisted (bystander-initiated) CPR^25^. Our results are in contrast to previous studies: time to first compression was comparable in V-CPR and T-CPR groups in one study^26^, and was significantly higher in V-CPR group in two studies^23,24^. In our study, connecting to the dispatcher was faster than in the mentioned studies in both V-CPR and T-CPR groups, however, it was in an optimal environment so probably it could take more time in a real-life OHCA situation.

In dispatcher-assisted CPR, human factors are essential, as well (e.g. trained dispatchers, physical abilities and attitudes of the lay responders)^27^. In our study, the self-assessment and attitudes were comparable between all of the groups. The vast majority of the participants had a positive attitude about the V-CPR technology in a possible real-life situation. In a prior study, dispatchers had also a positive attitude about the V-CPR, however, they stated that it does not result automatically an improvement in CPR quality^28^. For successful implementation, specific training of the dispatchers and lay responders are essential^12,27^.

Based on our results, V-CPR is feasible to improve the quality of CPR compared to T-CPR and U-CPR. However, analyzing the data from the view of real-life requirements (e.g. international guidelines), only a minority of the V-CPR participants were able to achieve high-quality chest compressions in all aspects together which conformed to the ERC guidelines^16^. This latter point indicates that the characteristics (e.g. physical abilities, prior training, prior experience, etc.) of the lay responders are also an important factor during V-CPR so this novel technology can only be influenced to a limited extent by the dispatcher so further studies are necessary focusing on this issue. Preparation may take a longer time using V-CPR technology, however, it can pay off over time because of the correction opportunity by the real-time feedback. As a psychological effect in the V-CPR group, being calmer during CPR could have positive effects on performance. In addition, a positive attitude can also be useful in implementation.

Our study has several limitations. First, since V-CPR is not been implemented in real emergency situations in Hungary yet, we could not set up a „real dispatcher room” fitting the requirements of V-CPR, and we did not have a standardized V-CPR protocol (dispatchers used the usual T-CPR protocol with some modifications expanded by the live video picture). Second, our study was made under optimal circumstances (sufficient light, stable WiFi connection, no negative environmental and weather factors, etc.), and we used a CPR manikin as the victim during a 2 min long scenario. In addition, participants were informed about the opportunities of the different groups which limited the realism. Regarding these facts, we do not know, how our participants would react in a possible real-life OHCA situation, or what would be the results during a longer scenario. Third, we used a study operator who handled the smartphone and managed the emergency call. In a real emergency situation, a second person may not always be present and/or is not prepared with the usage of the V-CPR technology. Furthermore, the participants in our study were university students (young adults). Examining different age groups of lay responders would lead to other results.

## Conclusion

We could not show any difference in chest compression depth comparing V-CPR with T-CPR and U-CPR groups in a simulation setting. However, V-CPR was superior to T-CPR and U-CPR in chest compression rate and correct hand position.

### Supplementary Information

Supplementary Figures.

## Data Availability

The datasets generated during and/or analysed during the current study are available from the corresponding author on reasonable request.
