# Supplementary material for: A randomized controlled simulation trial comparing video-assisted with telephone-assisted and unassisted cardiopulmonary resuscitation performed by non-healthcare university students
Source: Sci Rep. 2023 Sep 11;13:14925. doi: 10.1038/s41598-023-42131-z (PMC10495456; doi:10.1038/s41598-023-42131-z)
Supplement: Supplementary file 1 — Supplementary Figures. [file 41598_2023_42131_MOESM1_ESM.pdf]

Supplementary Material

Supplementary Figure S1. The English-translated version of the Hungarian T-CPR protocol. The protocol was shortened and modified to our study (e.g. no instructions about breathing patients, or no ask for an automated external defibrillator because these were not the focus of our study).

Is the patient lying on his/her back?

↓ YES

Kneel next to the patient. Put your one hand on the patient’s forehead and the other hand under the patient’s chin. Tilt the head back gently. Did it work?

↓ YES

Lean in to the patient’s face and check the movements of the chest and feel and hear the airflow for 10 seconds (I will count). Is the patient breathing normally? Did the patient have at least two normal breathing? Is it any movements (swallowing, caughing, hiccup)?

↓ NO

You have to start CPR. I will help you. If you are alone, shout for help immediatelly.

Kneel by the side of the patient. Place the heel of one hand in the centre of the patient’s chest which is the lower half of the sternum. Place the heel of your other hand on top of the first hand and interlock your fingers. Keep your arms straight. Press down the sternum at least 5 cm but no more than 6 cm. I will count, please keep the chest compression rate. After each compression, release the chest fully without losing the contact between your hand and the patient’s chest.

Is it working? Can you performing the chest compressions?

↓ YES

All right. Do it continuously, I will help you and count (counting every 30 seconds for 10 seconds).

Please immediatelly let me know if the patient is moving.

The ambulance is on way, keep performing chest compressions continuously.

Supplementary Figure S2. The English-translated version of the Hungarian T-CPR protocol was modified for the V-CPR technology. The protocol was shortened and modified to our study (e.g. no instructions about breathing patients, or no ask for an automated external defibrillator because these were not the focus of our study). In addition, the protocol was optimized for the V-CPR technology (e.g. the question „Is the patient laying on his/her back?" was not necessary in the V-CPR group because the dispatcher could see the patient's position without asking the lay responder).

Kneel next to the patient. Put your one hand on the patient's forehead and the other hand under the patient's chin. Tilt the head back gently. Did it work?

↓ YES

Lean in to the patient's face and check the movements of the chest and feel and hear the airflow for 10 seconds (I will count). Is the patient breathing normally? Did the patient have at least two normal breathing? Is it any movements (swallowing, caughing, hiccup)?

↓ NO

You have to start CPR. I will help you. If you are alone, shout for help immediatelly.

Kneel by the side of the patient. Place the heel of one hand in the centre of the patient's chest which is the lower half of the sternum. Place the heel of your other hand on top of the first hand and interlock your fingers. Keep your arms straight. Press down the sternum at least 5 cm but no more than 6 cm. I will count, please keep the chest compression rate. After each compression, release the chest fully without losing the contact between your hand and the patient's chest.

Is it working? Can you performing the chest compressions?

↓ YES

All right. Do it continuously, I will help you and count (counting every 30 seconds for 10 seconds and immediate feedback if something is wrong).

Please immediatelly let me know if the patient is moving.

The ambulance is on way, keep performing chest compressions continuously.
